# The Role of the Theory of Planned Behavior in Explaining the Energy-Saving Behaviors of High School Students with Physical Impairments

**DOI:** 10.3390/bs12090334

**Published:** 2022-09-15

**Authors:** Sirinakorn Suntornsan, Surapong Chudech, Piyapong Janmaimool

**Affiliations:** 1 Environmental Social Sciences Program, School of Liberal Arts, King Mongkut’s University of Technology Thonburi, 126 Pracha-Uthit Rd., Thungkru, Bangkok 10140, Thailand; 2 ASEAN Institute for Health Development, Mahidol University, 999 Salaya, Nakhon Pathom 73170, Thailand

**Keywords:** the theory of planned behavior, perceived behavioral control, attitude towards behaviors, subjective norm, students with physical impairments

## Abstract

People with physical impairments can help solve energy problems by participating in diverse energy-saving behaviors, such as switching off lights or turning off an air conditioner when not in use; however, they may struggle to participate in some behaviors due to mobility impairments. This study aims to examine factors that impact the energy-saving behaviors of high school students with physical impairments. The theory of planned behavior (TPB) was used to test whether attitudes towards energy-saving behaviors, subjective norms, and perceived behavioral control could affect intentions, which then leads to performance of energy-saving behaviors. The participants were 330 high school students with physical impairments in Thailand. A questionnaire was employed to measure energy-saving behaviors and TPB constructs. A confirmatory factor analysis (CFA) was performed to validate all study variables; structural equation modeling (SEM) was then used to test causal relationships among TPB constructs and energy-saving behaviors. The results showed that the TPB could be used to explain the energy-saving behaviors of students with physical impairments, that subjective norms were the most significant predictor of behavioral intentions, and that intentions significantly impacted energy-saving behaviors. While perceived behavioral control did not have a direct effect on behaviors, it had a significant effect on intentions. Under the TPB construct, of the studied variables, attitude had the lowest power to predict students’ intentions to perform the concerned behaviors; however, the impact of attitude was still statistically significant. The results suggest that all TPB variables can predict energy-saving behaviors of high school students with physical impairments, but their power to predict the behaviors is different. To promote student participation in energy-saving behaviors, it is important to create subjective norms and eliminate obstacles that students with physical impairments might face when performing energy-saving behaviors.

## 1. Introduction

Energy production and consumption contribute significantly to climate change [1,2], which is now considered a global environmental crisis. Solving this energy crisis requires the participation of all sectors, including business, agriculture, transportation, governments, and households, as well as of individuals. Individual participation in energy-saving behaviors is crucial, as it can reduce energy demand and minimize the adverse environmental impacts of energy production and consumption [3,4]. People can adopt energy-saving behaviors in various ways, including using energy-efficient appliances, implementing energy-efficient measures in buildings, adopting renewable energy sources and technologies, and practicing energy-reducing behaviors [5]. Jareemit and Limmeechokchai [6] reveal that participation in energy-saving behaviors could help reduce annual household energy use by 7–15%, or 484–1038 kWh. Furthermore, Steg [7] finds that the active engagement of individuals in SEBs helps to reduce CO_2_ emissions.

Currently, energy-saving behaviors are being widely promoted to solve energy-related problems. Many previous studies have explored strategies to enhance individual participation in energy-saving behaviors, but most of these focus on the participation of individuals with normal physical bodies [7,8,9]. However, people with physical impairments can also participate in energy-saving behaviors, and their participation in these behaviors can contribute to a great amount of energy saved. According to the World Health Organization [10], approximately 15% of the world’s population, or one billion people, experience some form of disability. In Thailand, there were approximately 2.1 million disabled people in 2021 [11], and half of this number (1,054,786) had physical impairments. Most importantly, the number of physically disabled people in Thailand is increasing due to the rise in the average age of the population and chronic health conditions. Therefore, the participation of people with physical impairments could significantly contribute to solving energy problems. Aside from contributing to solving the energy problem, promoting participation in energy-saving behaviors by people with physical impairments also enhances their self-esteem, as they could perceive their value to society.

However, physical impairments could hinder people from participating in energy-saving behaviors due to limited physical capacity and dexterity. For instance, the interior of buildings might not be designed so that people with physical impairments can conveniently access electrical appliances. People with physical impairments, therefore, need to make more effort to participate in energy-saving behaviors that require physical movements. These include turning off lights, switching off electrical devices, and unplugging appliances. As a result, the determinants of energy-saving behavior participation among people with physical impairments could differ from those among people without physical impairments. 

This study intends to explore factors that impact participation in energy-saving behaviors among people with physical impairments; specifically, it focuses on the behavior of high school students in Thailand. High school students are capable of thinking critically, ethically, and creatively about environmental and sustainability issues, and they are able to make informed decisions about how to respond to environmental problems. Most importantly, to promote pro-environmental behaviors in the long term, environmental attitudes and pro-environmental behaviors should be promoted at a young age [12,13]. Doherty and Clayton [14] indicate that young people are most receptive to changing their behaviors if they realize the importance and value of those behaviors. Ding et al. [15] and Sintov et al. [16] found that student engagement in energy-saving behaviors not only helps reduce energy demands, but also significantly impacts sustainable development, as students can help make their societies more environmentally sustainable. Students can set a good example for other groups in society. To encourage student participation in sustainable behaviors, Badea et al. [17] propose a focus on education to raise student awareness of sustainability and participation in sustainable behaviors. However, an understanding of the determinants of student participation in such behaviors could have implications for the development of educational strategies.

This study employed the theory of planned behavior (TPB), which has been widely used to explore the pro-environmental behaviors (PEBs) of several population groups [18,19], except for the physically disabled population, to investigate significant factors that could predict participation in energy-saving behaviors by high school students with physical impairments. According to the TPB, individuals decide to engage in a certain behavior based on a rational thought process. The decision to engage in a behavior is impacted by behavioral intention, and intention is affected by subjective norms, attitudes toward the behavior, and perceived behavioral control. Most importantly, the joint function of behavioral intention and perceived behavioral control could significantly influence a behavior. This means that individuals who intend to perform a certain behavior, and concurrently have perceived behavioral control, are likely to participate in a certain behavior.

Subjective norms are related to social cohesion [20] and can be defined as how given behaviors are perceived by other people who are important to an individual. Individuals tend to adopt a behavior if they perceive that participation in that behavior is socially accepted. Attitude towards the behavior refers to one’s perception of the value of the behavior. Individuals with positive attitudes towards a behavior are more likely to intend to participate in the behavior. Perceived behavioral control refers to an individual’s level of comfort with a particular behavior. In other words, behavioral control is an individual’s perception of the availability of resources, capability, and opportunities to participate in a particular behavior [19]. Individuals with a high level of behavioral control tend to engage in a given behavior. The TPB also states that behavioral control can directly affect participation in a behavior, and having both behavioral control and intentions concurrently, individuals can easily decide to engage in the behaviors. The TPB has been successfully applied to explain decisions to engage in various behaviors, including green purchasing behaviors [21,22], water conservation behaviors [23,24], waste management behaviors [25,26], and energy-saving behaviors [9,27,28]. Therefore, this study employs the TPB to investigate participation in energy-saving behaviors by students with physical impairments. 

The goal of this study was to use the TPB to investigate factors that contribute to participation in energy-saving behaviors by high school students with physical impairments. As stated above, the size of the physically disabled population in Thailand has been increasing, and the participation of this group in energy-saving behaviors not only helps to solve energy problems but also enhances the self-esteem of physically disabled people. This is because physically disabled people could perceive their value to society. This study also focused on high school students because their participation in energy-saving behaviors can be developed in the long run and can affect the motivation of other groups to participate in energy-saving behaviors. The objectives of this study were to examine how perceived behavioral control, attitudes toward energy-saving behaviors, and subjective norms affect student intentions to participate in ESBs, which then leads to the performance of ESBs. According to the TPB, the study also examined how a joint function of perceived behavioral control and behavioral intentions affects energy-saving behaviors. A structural equation model (SEM) will be used to test the effect of each variable on energy-saving behaviors and to measure the causal relationships among variables. The results of this study could have implications for the development of communication strategies to promote participation in energy-saving behaviors by students with physical impairments.

## 2. Literature Review

### 2.1. Energy-Saving Behaviors

An energy-saving behavior is an individual action that aims to reduce energy consumption and the negative environmental impacts of energy consumption and production [29]. Most energy-saving behaviors are performed in the residential sector. Several scholars suggest how individuals can participate in energy-saving behaviors in the residential sector. Firstly, individuals may adopt energy-efficient behaviors [30], including using energy-efficient appliances (including light bulbs and large appliances) and efficient use of electricity (such as cleaning electrical appliances to improve their efficiency or adjusting the temperature of air conditioners). Steg et al. [5] indicate that switching to energy-efficient appliances can significantly reduce household energy consumption. Secondly, individuals can reduce energy use through two types of electricity-consumption reduction behaviors. The first type is curtailment behaviors, which include reducing the length of showers, turning off lights when not in use, and unplugging appliances [30]. The second type is avoidance activities; these involve avoiding high-energy activities, such as drying laundry in a machine and showering in hot water [31]. Steg et al. [7] indicate that adopting or switching to energy-efficient technology can reduce energy use and CO_2_ emissions more effectively than energy consumption reduction behaviors. However, the frequency of behaviors is also important. Zografakis et al. [32] argue that to successfully reduce electrical consumption, individuals must make lifestyle changes.

### 2.2. Theory of Planned Behavior

The TPB, which was proposed by Ajzen in 1985 [18], is an extension of the theory of reasoned action (TRA) [33,34]. Both models explain that an individual’s decision to engage in a certain behavior is based on logical and reasoned thought processes. According to the TPB, a behavior is directly determined by an individual’s intention to engage in it, and intention is influenced by the way that an individual perceives the value of the behavior (attitude towards the behavior) [35], how significant others in the individual’s life view or think about the behavior (subjective norms), and the perception that the certain behavior is within the individual’s control (perceived behavioral control) [19]. Three significant factors that could affect behavioral intention and behavior are discussed in the subsections below. 

#### 2.2.1. Attitude towards the Behavior

Individuals construct behavioral beliefs based on their evaluation of the concerned behavior. These behavioral beliefs and attitudes can be favorable or unfavorable [36]. Individuals with a positive attitude towards a behavior are more likely to intend to perform the behavior. 

#### 2.2.2. Subjective Norms

According to the TPB, if individuals believe that important people in their life would approve of a certain behavior, they are more likely to intend to engage in the behavior. Nugier et al. [36] state that social influence or social pressure could induce negative moral emotions such as embarrassment, shame, and guilt. These emotions could affect an individual’s intention to perform a certain behavior.

#### 2.2.3. Perceived Behavioral Control

Perceived behavioral control is generated from an individual’s belief that they have sufficient resources, capability, and opportunities to perform a given behavior. According to Ajzen [20], perceived behavioral control refers to an individual’s judgement of the ease or difficulty of participating in a particular behavior. Similarly, Abrahamse [37] defines perceived behavioral control as an individual’s assessment of the factors that might facilitate or impede a certain behavior. Based on the TPB, perceived behavioral control can directly influence behavioral intention. Namely, individuals with high perceived behavioral control may have more intentions to participate in a certain behavior than those with low perceived behavioral control. Most importantly, individuals who intend to perform a certain behavior, and concurrently have perceived behavioral control, are likely to participate in a certain behavior. For instance, individuals with the same level of intention to engage in a behavior might have different levels of engagement due to different levels of perceived behavioral control. Similarly, an individual’s intention might not result in a behavior due to a lack of perceived behavioral control.

In sum, it can be concluded that perceived behavioral control, together with subjective norms and attitudes toward the behavior, can directly influence intentions. Perceived behavioral control and intentions then determine behavior. Previous studies have used TPB to explain an individual’s participation in a variety of behaviors, including health behaviors [38], resource conservation [39,40], and safe environmental practices [41,42,43,44].

## 3. Theoretical Framework

**Figure 1:**
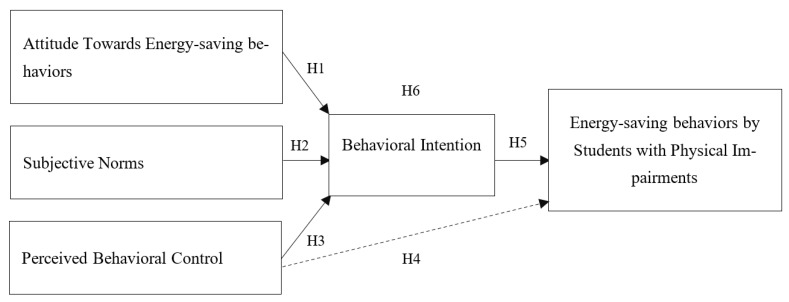
Theoretical Framework.

This study employs the TPB to investigate how students with physical impairments decide to participate in energy-saving behaviors. In this study, energy-saving behaviors refer to the participation of students with physical impairments in energy-saving behaviors in the residential sector. These energy-saving behaviors are behaviors that students with physical impairments can engage in. They include two types of energy-saving behaviors: curtailment behaviors (such as switching off appliances and lights when not in use) and avoidance behaviors (such as not taking hot showers in the summer and not using air conditioners in the winter). The TPB is used to explore factor affecting energy-saving behaviors. The TPB is most widely used for investigating the pro-environmental psychological domain [45]. Several previous studies have employed the TPB and related variables to investigate energy-saving behaviors [9,41,46,47], but no previous study has used the TPB to explore energy-saving behaviors among people with physical impairments. According to the TPB, factors that impact engagement in a given behavior include attitudes towards the behaviors, subjective norms, and perceived behavioral control. These three factors affect an individual’s behavioral intention, which finally leads to performance of the behavior [19]. In the current study, these three variables are assumed to significantly impact intention to perform Energy-saving behaviors and thus performance of energy-saving behaviors (see Figure 1). The study variables and research hypotheses are visualized in the figure below.

### 3.1. Attitudes towards Energy-Saving Behaviors

Attitudes towards energy-saving behaviors refers to an individual’s evaluation of energy-saving behaviors; it can be positive or negative. Several previous studies have found that attitude plays a significant role in determining energy-saving behaviors. Zhao et al. [48] report that attitude is the most influential factor impacting an individual’s intention to purchase energy-saving appliances. Attitude had a greater impact than social norms, perceived behavioral control, or moral norms. Wang et al. [49] report that intentions to save electricity were positively impacted by attitudes. Some studies have reported that attitude strongly predicts intention to purchase energy-efficient devices [50,51]. Olawale [52] also report that staff members’ intentions to perform energy-saving behaviors in a hotel are significantly impacted by attitude. Attitudes towards a behavior have also been reported to predict energy-saving behaviors in various counties [8,53]. Therefore, it may be assumed that attitude significantly impacts behavioral intention to perform energy-saving behaviors. Students with positive attitudes towards energy-saving behaviors could have a stronger intention to engage in energy-saving behaviors. Thus, the first hypothesis is:

**H1.**  *Attitude towards energy-saving behaviors positively impacts the intentions of students with physical impairments to perform energy-saving behaviors.*

### 3.2. Subjective Norms

Subjective norms refer to the perceived social pressure to engage in or social approval of energy-saving behaviors from people who are important to an individual. Individuals who perceive that others who are important to them approve of energy-saving behaviors are more likely to engage in energy-saving behaviors. Nie et al. [54] find that subjective norms are a powerful predictor of energy-saving behaviors in residential buildings. Goto et al. [55] report that energy-saving behaviors among university students in Vietnam are significantly affected by subjective norms. Several previous studies have found that subjective norms affect behavioral intention, which leads to performance of PEBs [56,57]. Luigina and Anna [58] find that the intentions of Italians to perform energy-saving behaviors were significantly impacted by subjective norms. Therefore, subjective norms can be assumed to positively impact intention to perform energy-saving behaviors, which leads to engagement in energy-saving behaviors. However, Du and Pan [9] find that subjective norms do not significantly impact energy-saving behaviors among students in Hong Kong. Thus, the second research hypothesis is:

**H2.**  *Subjective norms positively impact the intentions of students with physical impairments to perform energy-saving behaviors.*

### 3.3. Perceived Behavioral Control

Perceived behavioral control refers to an individual’s perception of their ability to engage in a given behavior. This perception is based on available resources and on the individual’s capabilities and opportunities to participate in energy-saving behaviors. According to the TPB, perceived behavioral control directly affects behavioral intention and performance of a behavior [19,59]. Therefore, individuals with high perceived behavioral control are more likely to have the intention to perform energy-saving behaviors and to participate in such behaviors. Many previous studies have found that perceived behavioral control significantly predicts intention to perform energy-saving behaviors [8,49,58,60]. Luigina and Anna [58] find that, of the three factors in the TPB, perceived behavioral control has the strongest predictive power of intention to perform energy-saving behaviors among Italian people. Du and Pan [9] also find that, of the three factors, perceived behavioral control has the strongest impact on the energy-saving intentions of students in Hong Kong. Setyawan et al. [61] find that the energy-saving behaviors of teenagers in Indonesia are significantly influenced by perceived behavioral control. Therefore, the third and fourth hypotheses are:

**H3.**  *Perceived behavioral control positively impacts the intention of students with physical impairments to perform energy-saving behaviors.*

**H4.**  *Perceived behavioral control positively impacts the participation of students with physical impairments in energy-saving behaviors.*

### 3.4. Behavioral Intention

Behavioral intention refers to an individual’s intent to perform energy-saving behaviors. According to the TPB, a decision to perform a behavior is directly determined by an individual’s intention to engage in it. Therefore, this study assumed that students with a higher level of intention to perform energy-saving behaviors are more likely to engage in these behaviors [62]. In addition, the TPB states that an individual’s participation in a certain behavior is positively influenced by a combination of intention and perceived behavioral control. Namely, behavioral intention can be a good predictor of behavior, only when individuals have control over behavioral performance [59]. Thus, intention could have a mediation effect on the relationship between perceived behavioral control and energy-saving behaviors as well. Therefore, the fifth and sixth hypotheses are:

**H5.**  *Intention to perform energy-saving behaviors positively impacts the participation of students with physical impairments in energy-saving behaviors.*

**H6.**  *Intention to perform energy-saving behaviors mediates the effect of perceived behavioral control on the energy-saving behaviors of students with physical impairments.*

## 4. Methods

### 4.1. Research Tool

This study used a questionnaire to collect data. Self-reports were used to measure the participants’ energy-saving behaviors [63]. Participants were asked about the frequency with which they engage in energy-saving behaviors; responses used a 7-point Likert scale ranging from 1, “never” to 7, “regularly” [64]. To measure variables that, based on the TPB, were assumed to impact energy-saving behaviors, the study also used 7-point Likert scale questions. The responses ranged from 1, “completely disagree” to 7, “completely agree.” To measure participation in energy-saving behaviors, 6 items were developed based on the recommendations of the Energy Policy and Planning Office (EPPO) [65] on individual participation in energy-saving behaviors and household electricity use reduction. To measure the TPB factors, items were developed based on the TPB [66]. Ten items were used to measure the participants’ attitudes towards energy-saving behaviors, 11 items to measure subjective norms, and 8 items to measure perceived behavioral control. Four items were used to measure behavioral intention (see Appendix A). All items in each construct were evaluated for validity and reliability before the data were analyzed.

### 4.2. Participants

The participants in this study were high school students with physical impairments in Thailand. A random simple sampling technique was used to select participants from 6 schools that particularly provide formal education for students with physical impairments. A total of 400 questionnaires were distributed to the participants; 356 questionnaires were returned. Of these, 26 returned questionnaires were excluded due to incompletion. A total of 330 questionnaires, or 82.5% of the distributed questionnaires, were included in the analysis. This comprises more than 200 participants, which is an appropriate number for an SEM analysis [67,68]. Before data collection, participants were informed of the study objectives. They were also informed that no negative impacts from participating in the survey were expected. All participants provided written consent to participate in the study, and the study was approved by the Research Ethics Committee of the School of Liberal Arts, King Mongkut’s University of Technology Thonburi (SoLA-EA-2021-0-003) on 26 March 2021.

### 4.3. Data Collection and Analysis

Before data were collected, the content validity of the questionnaire was evaluated. Three experts were invited to evaluate content validity for all factors. Some minor changes to some items were recommended to enhance the clarity of the items for high school students. The modified questionnaire was then sent to 30 students as a pilot test. These students were not participants in this study but had similar characteristics to the participants. Reliability was then measured using Cronbach’s alpha (α); for the entire questionnaire, Cronbach’s α was 0.71. This is greater than 0.70, indicating that the items are reliable [69]. Questionnaire surveys were conducted in 6 schools that provide formal education for students with physical impairments from April to May 2021. 

To analyze the data, a confirmatory factor analysis (CFA) was first performed to test the construct validity and discriminant validity of the scales [70]. Based on the CFA, the factor loading for each item in each variable was calculated. Items with factor loading values above 0.60 were used to measure the study variables. The internal consistency and reliability of each variable was also examined [71]. Secondly, an SEM was used to test the causal relationships among variables [70]. Energy-saving behavior engagement was defined as an endogenous variable that was impacted by the exogenous variables of behavioral intention and perceived behavioral control. Behavioral intention was also defined as an endogenous variable that was impacted by the exogenous variables of attitude toward energy-saving behaviors, subjective norms, and perceived behavioral control. Based on the SEM, the overall model fit was measured by calculating the following indexes: the model chi-square (X^2^), the ratio of chi-square to the degree of freedom (X^2^/df), the standardized root mean square residual (SRMR), the normed fit index (NFI), the Tucker–Lewis index (TLI), the comparative fit index (CFI), the goodness-of-fit index (GFI), and the incremental fit index (IFI) [72,73]. SPSS 22.0 and Amos 23 were used to perform the data analyses.

## 5. Results

### 5.1. Participant Characteristics

Participants in this study were students with physical impairments from six schools in Thailand. More than half of the participants were female students (54.20%) and 45.80% of participants were male. Almost half (44.24%) of participants were 14 years old or younger; 45.75% were aged 15 to 17 years. Only 10% of participants were 18 years old or older. The majority of participants were in junior high school (see Table 1).

### 5.2. Measurement Model

A confirmatory factor analysis (CFA) was conducted to evaluate the fitness of the measurement model to the data before the relationships among the TPB variables and energy-saving behaviors were evaluated. The CFA showed that the measurement model has a good fit (*p* < 0.001, χ^2^/df = 1.63, IFI = 0.99, TLI = 0.99, NFI = 0.99, GFI = 0.99, RMSEA = 0.04, CFI = 0.99). All the indices revealed that the measurement model was acceptable, particularly when items with low factor loading values (<0.50) were excluded from the CFA [67] to decrease measurement errors (see Table 2). The factor loadings of all items shown in Table 2 were above the standard value, indicating convergent validity. After the CFA analysis, Cronbach’s α coefficients were calculated to evaluate the internal reliability of the measures. Cronbach’s α coefficients for the scales ranged from 0.74 to 0.84; all were above the threshold of 0.7 [74]. In addition, to test the convergence validity of the different latent variables, average variance extraction (AVE) scores were calculated. The AVE scores ranged from 0.511 to 0.84, greater than the generally accepted minimum requirement of 0.5 [75]. Finally, the AVE scores were greater than the correlations between the study variables (see Table 3) [76], indicating discriminant validity. 

Table 3 shows the average score for each variable and the correlations among them. Most of the correlation coefficients for the TPB variables and energy-saving behaviors were statistically significant. This implies that there may be associations among the variables. The coefficient values were lower than 0.60, indicating that no multicollinearity problem was present [77].

### 5.3. Effects of TPB Variables on Energy-Saving Behaviors

To test the effects of the TPB variables on energy-saving behaviors, an SEM was used. Firstly, the overall fit of the model to the data was checked; the fit was acceptable. The value of χ^2^ was not statistically significant (χ^2^ = 0.615, df = 2, probability level = 0.735), and the ratio of chi-square/degree of freedom was (χ2/df) 0.31, which is lower than 5.0. It implies that there was a close fit between the proposed model and the observed data [78]. The results of the model assessment showed a statistically acceptable GFI value of 0.99. The GFI value, which is greater than 0.90, can indicate a close fit between the data and the proposed model [78]. The root means square error of approximation (RMSEA) had a value of 0.00, which is less than 0.08. This implies that the model is a reasonable approximation of the data. As suggested by Brown and Cudeck [79], an acceptable value of RMSEA that indicates a reasonable error of approximation must be lower than 0.08. The comparative fit index (CFI) value, used to indicate the discrepancy function adjusted for sample size, was also statistically acceptable, CFI = 1.00. Hu and Bentler [80] suggest that an acceptable model fit should have a CFI value of 0.90 or larger. Additionally, the incremental fit index (IFI), used to indicate the possibility of having the worst model, had a value of 1.00, which is greater than 0.900, thus indicating the acceptability of the proposed model [81]. Other important indexes are the normed fit index (NFI) and the Tucker–Lewis index (TLI). As suggested by Meyers et al. [81], values of 0.7 and greater for these indices indicate satisfactory fit, 0.8 and greater reveal fit well, and 0.9 and greater show fit perfectly. In this study, NFI of 0.99 and TLI of 1.00 demonstrated the model fit. Overall, it can be summarized that the data fits the model, and the proposed model is statistically acceptable. 

**Figure 2:**
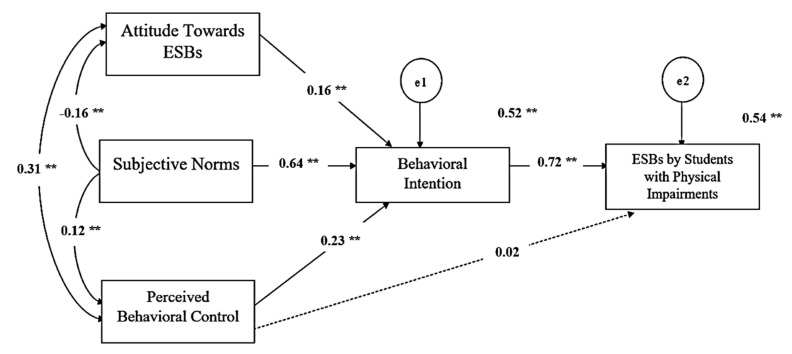
The structural equation modeling (SEM). (Note: ** *p* < 0.001).

The effects of each variable on energy-saving behaviors were then examined by considering the path coefficients among the variables. The result indicated a squared multiple correlations value of 0.54. This means that approximately 54% of the variance in energy-saving behaviors could be accounted for by the linear combination of all TPB variables, which include attitude towards energy-saving behaviors, subjective norms, perceived behavioral control, and behavioral intention (see Figure 2). Hypotheses one to three state that attitude towards energy-saving behaviors (β = 0.16, *p* < 0.01), subjective norms (β = 0.64, *p* < 0.01), and perceived behavioral control (β = 0.23, *p* < 0.01) significantly impact behavioral intention, or the intention to perform energy-saving behaviors. Therefore, hypotheses one to three were accepted. However, calculating the power of effect (β) showed that subjective norms had the strongest impact on behavioral intention. The predicted path from perceived behavioral control to energy-saving behaviors was not statistically significant (β = 0.02, *p* > 0.05). Therefore, hypothesis four was not accepted. Energy-saving behaviors were positively and significantly impacted by behavioral intention (β = 0.72, *p* < 0.001); therefore, hypothesis five was accepted. Finally, although perceived behavioral control had no significant impact on energy-saving behaviors, behavioral intention significantly mediated the relationship between perceived behavioral control and energy-saving behaviors (β = 0.26, *p* < 0.05). Therefore, hypothesis six was accepted (see Table 4).

Of the studied variables, subjective norms had the strongest impact on behavioral intention, followed by perceived behavioral control (see Table 5). Attitude towards energy-saving behaviors had the weakest impact. Behavioral intention had a strong direct effect on energy-saving behaviors, while subjective norms had a strong indirect effect on energy-saving behaviors through behavioral intention.

## 6. Discussion and Conclusions

This study has investigated determinants of energy-saving behaviors among high school students with physical impairments. Students with physical impairments include persons with poor manual dexterity, lower limb loss or impairment, and/or damage to one or multiple organs of the body. Due to their mobility impairments, these students are likely to find participating in energy-saving behaviors more difficult than those with normal physical bodies. This study employed the TPB to explore how these students decide to perform energy-saving behaviors. An SEM was used to test the causal relationships among variables. The results revealed that TPB constructs accounted for a large proportion (54%) of variance in the engagement of high school students in energy-saving behaviors. This confirmed that the TPB can be used to explain energy-saving behaviors among students with physical impairments. De Leeuw et al. [82] also found that many TPB constructs could predict the engagement of high school students in PEBs. However, De Leeuw et al. [82] found that perceived behavioral control was the strongest predictor of behavioral intention, while the current study found that subjective norms were the strongest predictor of intention to perform energy-saving behaviors. This result aligns with those of Anderson [83], who found that, among TPB variables, subjective norms had the strongest positive impact on powerful variable behavioral intentions and PEBs among university students in the United States. Similarly, Gusmerotti et al. [84] found that subjective norms were the strongest predictor of high school students’ PEBs related to marine litter in Italy, while attitude had a limited impact. Halder et al. [85] found that subjective norms had the second-strongest positive impact on high school students’ intentions to use bioenergy. Chen and Knight [47] also found that energy-saving behaviors were strongly predicted by subjective norms. 

In the present study, students with physical impairments intended to perform energy-saving behaviors if most people who were important to them also participated in energy-saving behaviors or encouraged them to practice the behaviors. Therefore, engagement in energy-saving behaviors by people who are important to students with physical impairments can significantly impact their intentions and motivation to participate in energy-saving behaviors. It can be recommended that high schools set up rules and actively adopt a pro-environmental identity so that teachers and staff will perceive energy-saving practices as part of their job duties. When students realize that energy-saving behaviors are generally engaged in by teachers and staff in a school, they are more likely to participate in the behaviors too. The results of this study demonstrated that subjective norms had a strong and direct impact on behavioral intention and that intention had a very strong impact on energy-saving behaviors. Additionally, students with physical impairments should be informed that energy-saving behaviors should be practiced by all groups of people and that people with physical impairments are not exempted. This is because environmental and resource conservation should be the moral responsibility of all groups in society. Furthermore, to promote participation in energy-saving behaviors, it is also important to enable students with physical impairments to realize that participation in energy-saving behaviors is socially accepted and that engaging in energy-wasting behaviors can be negatively viewed.

Perceived behavioral control also significantly affected the intention to perform energy-saving behaviors among students with physical impairments. Some previous studies have identified perceived behavioral control as the strongest predictor of intention to perform sustainable behaviors [73,77]. For instance, De Leeuw et al. [82] found that perceived behavioral control was the strongest predictor of intention to perform PEBs among high school students in Luxembourg. Similarly, Macove [86] found that perceived behavioral control was the strongest predictor of intention to perform energy-saving behaviors among individuals in Romania. In contrast, Swaim [87] found that perceived behavioral control was a poor predictor of students’ sustainable behaviors.

The current study confirms the role of perceived behavioral control in promoting behavioral intentions and energy-saving behaviors among students with physical impairments. Enhancing students’ feelings of control can encourage engagement in energy-saving behaviors. This type of student may struggle to engage in energy-saving behaviors due to mobility impairments. To promote their engagement in energy-saving behaviors, obstacles must be eliminated, and their capability to engage in energy-saving behaviors should be promoted through supportive environments. The present study found that perceived behavioral control did not directly impact energy-saving behaviors, but behavioral intention mediated the effect of perceived behavioral control on behaviors. This result is supported by the TPB [19], which indicates that intentions mediate the relationship between perceived behavioral control and specific behaviors. This means that the combination of perceived behavioral control and behavioral intention could significantly increase energy-saving behaviors among students with physical impairments.

To enhance energy-saving intentions and behaviors among students with physical impairments, it is important to increase their perceived behavioral control. In this way, it can be suggested that providing proper electricity control features that are easy to use for students with physical impairments is very important. For instance, the locations of light switches in a building should be convenient for people with physical impairments. In addition, building environmental control features, such as thermostat controls, must be designed in a way that is easy to use so that students can make choices in using those building environmental control features based on their environmental awareness. Most importantly, there are some social pressures that could hinder students from participating in energy-saving behaviors [88]. For instance, students may not be allowed to turn on/off electricity devices in a school without permission from a teacher, so they can hardly participate in energy-saving behaviors. In another case, students may not be allowed control over building environments without permission from the responsible staff, so their participation in energy-saving behaviors can be limited. For instance, Ackerly et al.’s study [89] shows that if there are automated notices on the windows telling the staff that it is acceptable to open or close the window, the staff become less concerned about the negative feedback of others. So, the staff can open the window instead of turning on the air conditioner when the weather outside is good. Therefore, high schools should provide students with proper physical building environments that are friendly to people with physical impairments and with electrical control features that are easy for this group of students to use. In addition, social pressures related to accessibility to a building’s control features must be avoided by providing messages that give students the authority to exercise control over the building environments. 

The current study also found that of the studied variables, students’ attitudes toward energy-saving behaviors had the smallest effect on both behavioral intention and participation in energy-saving behaviors. Similarly, De Leeuw et al. [82] found that of the studied variables, attitude had the least power to predict high school students’ intentions to perform pro-environmental behaviors [82]. Gusmerotti et al. [84] also found that attitude had a limited impact on high school students’ PEBs related to marine litter in Italy. In contrast, Swaim [87] found that, among TPB constructs, attitude had the strongest influence on college and university students’ intentions to engage in behaviors promoting environmental sustainability. The findings of the present study indicate that high school students with physical impairments decided to engage in energy-saving behaviors based primarily on subjective norms and perceived behavioral control. However, the effect of attitude on intention was still statistically significant. Therefore, the role of attitude should not be neglected. To create a positive attitude toward energy-saving behaviors among students with physical impairments, the students should be educated on the potential benefits of energy-saving behaviors and on the values of energy conservation. Schools can improve student perception of the benefits of individual participation in energy-saving behaviors. This can be done by showing students how much energy and money they can save for the school or their house if they take part in energy-saving behaviors. The attitude toward energy-saving behaviors depends on the evaluation of the outcome of the behavior, whether it is beneficial or pleasant. Some people may wrongly evaluate the outcomes of energy-saving behaviors, which negatively affects their attitudes toward such behaviors. For instance, some students may think that merely switching off the light for an hour in a year cannot really contribute to energy saving. However, if many people do it together following the same pattern, a great amount of energy can be saved. Therefore, this study suggests that students should be educated on the potential outcomes of each energy-saving behavior to create a positive attitude toward the behaviors.

The present study also found that behavioral intention significantly impacted student engagement in energy-saving behaviors. This result is consistent with many previous studies that used the TPB to investigate PEBs [57,77,90,91]. Therefore, when students’ intentions to perform energy-saving behaviors are enhanced, they are more likely to participate in energy-saving behaviors. As discussed above, efforts to enhance these intentions should focus on subjective norms and perceived behavioral control. Regarding the theoretical implications of the present study, it can be suggested that in predicting participation in energy-saving behaviors among high school students with physical impairments, TPB variables, all together, can have a strong power to predict the behaviors. Among the three TPB variables that are assumed to influence behavioral intention, subjective norms have the strongest effect on behavioral intention and energy-saving behaviors. Several studies that focused on the pro-environmental behaviors of normal high school students [84] and college students [92], not particularly those with physical impairments, also found that subjective norms were the strongest predictor of pro-environmental behaviors, including energy saving. This could imply that the decision to engage in pro-environmental behaviors among high school students or young people with or without physical impairments relies heavily on social influence. However, students’ physical impairments could negatively affect perceived behavioral control. This study found that perceived behavioral control had the second-strongest positive impact on high school students’ intentions to participate in energy-saving behaviors. It can be assumed that high school students with physical impairments are likely to face difficulties in performing energy-saving behaviors. For instance, improperly designed interior infrastructures that are not easy to use for people with physical impairments may contribute to hesitation to participate in energy-saving behaviors. Attitude had the weakest power in predicting both behavioral intention and participation in energy-saving behaviors. This implies that having only a positive attitude toward energy-saving behaviors might not be sufficient to promote participation in energy-saving behaviors among high school students with physical impairments.

In conclusion, the results of this study suggest that subjective norms and perceived behavioral control play important roles in enhancing intentions to perform energy-saving behaviors among high school students with physical impairments. The practices of people who are important to participants, such as teachers, family members, and friends, can significantly motivate this group of students to take part in energy-saving behaviors. Most importantly, obstacles that may hinder such students’ engagement in energy-saving behaviors should be eliminated. These may include interior building designs that make it difficult for people with physical impairments to access electrical appliances. Difficulty using or accessing electrical devices may prevent students with physical impairments from participating in energy-saving behaviors. Compared to other TPB constructs, the power of attitude towards energy-saving behaviors to predict behavioral intentions is not that significant, but this factor should not be overlooked. Positive attitudes towards energy-saving behaviors should be promoted among students with physical impairments through education about energy-saving behaviors and their potential benefits. 

## 7. Limitation of the Study and Future Research

There are some limitations which need to be addressed. Firstly, most participants in this study were junior high-school students. The result might not be generalizable for all high school students. Another potential limitation of this present study is its reliance on self-reports of energy-saving behaviors rather than observations and the possibility that participants might have over-estimated the extent to which they performed these behaviors. However, other studies related to pro-environmental behaviors mostly employed self-reports of pro-environmental behaviors. Thus, this study can be comparable to most other related studies. However, it can be recommended that future research should adopt observations on actual participation in pro-environmental behaviors. This can enhance credibility and trustworthiness of research findings.

Moreover, in this study, the TPB model predicted 54.9% of energy-saving behaviors. There are some variables related to socio-economic characteristics of participants which might be also associated with energy-saving behaviors, but this study did not take them into consideration. Further studies that can include socio-economic characteristics of participants, such as age and education level, are recommended. In addition, future studies that could extend the TPB model by including additional internal variables, such as personal norm, environmental awareness, and environmental knowledge, are recommended. 

## Figures and Tables

**Table 1:** Participant characteristics (n = 330).

Demographic Characteristics		Number	Percentage (%)
Gender	Female	179	54.20%
	Male	151	45.80%
Age	≤14 years old	146	44.24%
	15–17 years old	151	45.75%
	≥18 years old	33	10%
School Level	Junior high school	271	82.12%
	Senior high school	59	17.87%

**Table 2:** Results of confirmatory factor analysis.

Construct	Item	Mean	SD	Standardized Factor Loading	Cronbach’s Alpha	AVE
Attitude Towards Energy-saving behaviors	A2	5.20	0.58	0.88 ***	0.83	0.71
	A3	5.21	0.56	0.87 ***		
	A4	5.68	0.65	0.80 ***		
	A5	5.35	0.63	0.81 ***		
Subjective Norms	S1	6.50	0.74	0.94 ***	0.84	0.84
	S5	6.53	0.72	0.97 ***		
	S7	6.51	0.72	0.96 ***		
	S8	6.50	0.74	0.78 ***		
Perceived Behavioral Control	P1	6.48	0.61	0.92 ***	0.82	0.81
	P3	6.58	0.65	0.95 ***		
	P5	6.56	0.65	0.83 ***		
	P6	6.55	0.69	0.89 ***		
Behavioral Intention	B1	6.65	0.55	0.68 ***	0.74	0.51
	B2	6.54	0.82	0.86 ***		
	B3	6.13	0.96	0.63 ***		
	B4	6.42	0.76	0.65 ***		
Energy-saving behaviors	E2	6.13	0.82	0.78 ***	0.76	0.54
	E3	6.62	0.56	0.77 ***		
	E4	6.55	0.79	0.73 ***		
	E5	6.10	0.94	0.66 ***		

*** *p* < 0.001.

**Table 3:** Results of correlation analysis.

Construct	Mean	SD	A	S	P	B	E
A: Attitude Towards Energy-saving behaviors	5.36	0.61	**0.84**				
S: Subjective Norms	6.51	0.73	−0.16 **	**0.91**			
P: Perceived Behavioral Control	6.54	0.78	0.31 **	0.12 *	**0.90**		
B: Behavioral Intention	6.44	0.77	0.13 **	0.54 **	0.36 **	**0.66**	
E: Energy-saving behaviors	6.35	0.78	0.11	0.45	0.28	0.60 **	**0.74**

(1) The diagonal (bold) elements are the square roots of AVE values, and the off-diagonal elements are the correlations among the constructs. (2) * *p* < 0.05 and ** *p* < 0.01.

**Table 4:** Mediation test using bootstrapping.

Path	Bootstrapping		95% Bias-Corrected CI		P
	Indirect Effect	Boot S.E.	Boot LLCI	Boot ULCI	
P→B→E	0.263	0.073	0.119	0.409	0.001

P = perceived behavioral control, B = behavioral intention, E = energy saving behaviors.

**Table 5:** Direct, indirect, and total effects of exogenous variables on endogenous variables.

Exogenous Variables	Behavioral Intention			Energy-Saving Behaviors		
	DE	IE	TE	DE	IE	TE
A: Attitude towards Energy-saving behaviors	0.162	-	0.162	-	0.117	0.117
S: Subjective Norms	0.639	-	0.639	-	0.463	0.463
P: Perceived Behavioral Control	0.233	-	0.233	0.022	0.169	0.191
B: Behavioral Intention	-	-	-	0.725	-	0.725

DE = direct effect, IE = indirect effect, TE = total effect.

## Data Availability

The data used to support the findings of current study are available from the corresponding author upon request.

## Appendix A. Questionnaire items

**Variable**	**Question**	**Mean**	**SD**
Energy- saving behaviors	E1: I switch off the lights even if I’m not using them for a short time.	6.12	0.82
	E2: Even if I didn’t turn on the lights or electrical appliances, I switch them off when they are not in use.	6.13	0.82
	E3: I normally switch the computer to sleep mode if I will not be using it for at least fifteen minutes.	6.62	0.56
	E4: If the weather is good, I open the windows instead of turning on an air conditioner or a fan.	6.55	0.79
	E5: I do not switch on the lights if natural light is sufficient.	6.10	0.94
	E6: I unplug my mobile phone charger before going to bed.	6.31	0.54
Attitude towards Energy Saving Behaviors	A1: My active participation in energy-saving behaviors is a good thing.	5.50	0.66
	A2: My active participation in energy-saving behaviors at school could help reduce electricity costs.	5.20	0.58
	A3: My active participation in energy-saving behaviors could help our country minimize energy demand and expenditure.	5.21	0.56
	A4: My active participation in energy-saving behaviors could help reduce my household electricity costs.	5.68	0.65
	A5: My active participation in energy-saving behaviors sets a good example for other people.	5.35	0.63
	A6: My active participation in energy-saving behaviors helps me feel disciplined and proud of myself.	5.52	0.63
	A7: Active participation in energy-saving behaviors is a waste of time.	5.50	0.90
	A8: Active participation in energy-saving behaviors is troublesome and makes me feel stressed.	5.55	0.84
	A9: Active participation in energy-saving behaviors makes me feel bored.	5.44	0.88
	A10: My active participation in energy-saving behaviors can make other people proud of me and earn their praise.	5.46	0.68
Subjective Norm	S1: My class teacher thinks that I should participate in energy-saving behaviors.	6.50	0.74
	S2: My parents think that I should participate in energy-saving behaviors.	6.48	0.78
	S3: My close friends think that I should participate in energy-saving behaviors.	6.16	0.75
	S4: My classmates think that I should participate in energy-saving behaviors.	6.35	0.69
	S5: My class teacher actively participates in energy-saving behaviors.	6.53	0.72
	S6: My parents actively participate in energy-saving behaviors.	6.53	0.74
	S7: My close friends actively participate in energy-saving behaviors.	6.51	0.72
	S8: My classmates actively participate in energy-saving behaviors.	6.50	0.74
	S9: Most people actively participate in energy-saving behaviors.	6.16	0.57
	S10: Most people who are important to me would agree that I actively participate in energy-saving behaviors.	6.41	0.76
	S11: Most people who are important to me encourage me to actively participate in energy-saving behaviors.	6.30	0.77
Perceived Behavioral Control	P1: Despite my physical impairments, I can actively participate in energy-saving behaviors.	6.48	0.61
	P2: My physical impairments create an obstacle to my participation in energy-saving behaviors.	6.25	1.40
	P3: I am confident that I can actively participate in energy-saving behaviors.	6.58	0.65
	P4: My determination to perform energy-saving behaviors drives my participation in energy-saving behaviors.	6.61	0.57
	P5: It is entirely up to me whether I participate in energy-saving behaviors or not.	6.56	0.65
	P6: Even if I’m running late, I still check that all lights and electrical appliances are switched off before leaving home.	6.55	0.69
	P7: If I am exhausted, it is more difficult for me to participate in energy-saving behaviors.	6.18	1.28
	P8: If I need a lot of time to do my homework, it is more difficult for me to participate in energy-saving behaviors.	6.28	0.98
Behavioral Intention	B1: I intend to participate in energy-saving behaviors in my daily life.	6.65	0.55
	B2: I intend to switch off lights and electrical appliances before leaving home.	6.54	0.82
	B3: I will not leave the television or computer on while I am sleeping.	6.13	0.96
	B4: If my school holds a campaign to promote energy-saving behaviors, I intend to participate in energy-saving behaviors in school.	6.42	0.76
